# The IMpact of PerioperAtive KeTamine on Enhanced Recovery after Abdominal Surgery (IMPAKT ERAS): protocol for a pragmatic, randomized, double-blinded, placebo-controlled trial

**DOI:** 10.1186/s12871-023-02177-y

**Published:** 2023-06-30

**Authors:** Britany L. Raymond, Brian F. S. Allen, Robert E. Freundlich, Crystal G. Parrish, Jennifer E. Jayaram, Jonathan P. Wanderer, Todd W. Rice, Christopher J. Lindsell, Kevin H. Scharfman, Mary L. Dear, Yue Gao, William D. Hiser, Matthew D. McEvoy, for the Vanderbilt Learning Healthcare System Platform Investigators, Gordon R. Bernard, Robert S. Dittus, Shon Dwyer, Peter J. Embí, Chad Fitzgerald, Cheryl L. Gatto, Frank E. Harrell, Paul A. Harris, Tina Hartert, Jim Hayman, Catherine H. Ivory, Ruth Kleinpell, Sunil Kripalani, Lee Ann Liska, Patrick Luther, Jay Morrison, Thomas Nantais, Jill M. Pulley, Kris Rehm, Russell L. Rothman, Patti Runyan, Wesley H. Self, Matthew W. Semler, Robin Steaban, Cosby A. Stone, Philip D. Walker, Consuelo H. Wilkins, Adam Wright, Autumn D. Zuckerman

**Affiliations:** 1 Department of Anesthesiology, Vanderbilt University Medical Center, 1301 Medical Center Drive 4648 TVC, Nashville, TN 37232 USA; 2 Vanderbilt Institute for Clinical and Translational Research, Vanderbilt University Medical Center, 2525 West End Ave Ste. 600, Nashville, TN 37203 USA; 3 Department of Pharmaceutical Services, Vanderbilt University Medical Center, 1211 Medical Center Drive B-101, Nashville, TN 37232 USA

**Keywords:** Enhanced recovery pathways, Enhanced recovery after surgery, ERAS, Ketamine, Length of stay, Multimodal analgesia

## Abstract

**Background:**

Multimodal analgesic strategies that reduce perioperative opioid consumption are well-supported in Enhanced Recovery After Surgery (ERAS) literature. However, the optimal analgesic regimen has not been established, as the contributions of each individual agent to the overall analgesic efficacy with opioid reduction remains unknown. Perioperative ketamine infusions can decrease opioid consumption and opioid-related side effects. However, as opioid requirements are drastically minimized within ERAS models, the differential effects of ketamine within an ERAS pathway remain unknown. We aim to pragmatically investigate through a learning healthcare system infrastructure how the addition of a perioperative ketamine infusion to mature ERAS pathways affects functional recovery.

**Methods:**

The IMPAKT ERAS trial (IMpact of PerioperAtive KeTamine on Enhanced Recovery after Abdominal Surgery) is a single center, pragmatic, randomized, blinded, placebo-controlled trial. 1544 patients undergoing major abdominal surgery will be randomly allocated to receive intraoperative and postoperative (up to 48 h) ketamine versus placebo infusions as part of a perioperative multimodal analgesic regimen. The primary outcome is length of stay, defined as surgical start time until hospital discharge. Secondary outcomes will include a variety of in-hospital clinical end points derived from the electronic health record.

**Discussion:**

We aimed to launch a large-scale, pragmatic trial that would easily integrate into routine clinical workflow. Implementation of a modified consent process was critical to preserving our pragmatic design, permitting an efficient, low-cost model without reliance on external study personnel. Therefore*,* we partnered with leaders of our Investigational Review Board to develop a novel, modified consent process and shortened written consent form that would meet all standard elements of informed consent, yet also allow clinical providers the ability to recruit and enroll patients during their clinical workflow. Our trial design has created a platform for subsequent pragmatic studies at our institution.

**Trial registration:**

NCT04625283, Pre-results.

## Background

A multimodal approach to perioperative analgesia is recommended in clinical guidelines and is essential to Enhanced Recovery After Surgery (ERAS) clinical practice [1, 2]. This analgesic strategy encourages a synergism between different classes of medications to reduce the overall consumption of any single agent, especially opioids, thus minimizing dose-related side effects and accelerating recovery. Various protocols are well-supported within the literature. However, the individual contributions of each analgesic agent, both positive and negative, to the overall combined effects remains unknown. Thus, the optimal analgesic regimen that enhances recovery and limits side effects has yet to be established [1].

Ketamine is a non-narcotic analgesic that antagonizes the N-methyl-D-aspartate (NMDA) receptor to produce a reduced response to noxious stimuli [3]. Significant evidence supports the efficacy of subanesthetic doses of ketamine in the treatment of acute post-surgical pain [2–4]. Compared to placebo, or as an addition to opioid-based analgesia, the administration of perioperative intravenous ketamine has been shown to improve the subjective, patient-reported quality of analgesia while reducing opioid requirements by 30–50%, with particular benefit in abdominal, thoracic, and orthopedic surgeries [4, 5]. Prolonged infusions that continue into the postoperative period are superior to single bolus administrations [5], as the maintenance of consistent plasma levels during recovery can reduce the response to noxious stimuli that occurs with mobilization [6, 7].

Ketamine expresses unique pharmacodynamic properties, including the maintenance of respiratory and cardiovascular stability [3, 8, 9]. It has been associated with the treatment and prevention of chronic pain through the reduction of hyperalgesia and central sensitization [10]. Furthermore, NMDA agonism may play a role in neuronal modification, as ketamine therapy has shown promising improvement in the severity of psychiatric conditions such as refractory depression, post-traumatic stress disorder, and substance use disorders [11]. Even short-term exposures to ketamine around the time of surgery have been shown to improve mood and perception of pain postoperatively [12, 13]. These multiple factors have contributed to the growing popularity of ketamine as a multimodal adjunct in the perioperative setting. However, the magnitude of its effect within an established ERAS pathway remains debatable, as its effects on the pain experience and opioid consumption may be reduced in the setting of a multimodal analgesic ERAS regimen that includes regional anesthesia and other non-opioid multimodal analgesics. Thus, the potential benefit of ketamine to further reduce opioid consumption and speed recovery may be offset by its own side effect profile, which might inhibit functional recovery rather than promote it.

## Study objectives

The IMPAKT ERAS trial (IMpact of PerioperAtive KeTamine on​ Enhanced Recovery from Abdominal Surgery) will investigate the differential effect of ketamine infusions on recovery from major abdominal surgery within a standardized ERAS protocol. The primary outcome will be hospital length of stay (LOS), which is a customary marker of functional recovery for ERAS pathways. We hypothesize that LOS will be significantly reduced by the inclusion of perioperative ketamine infusions as part of the ERAS protocol in patients undergoing major abdominal surgery.

## Methods and analysis

### Study design

The IMPAKT ERAS trial was approved by Investigational Review Board (IRB, approval number 200210) at Vanderbilt University Medical Center (VUMC), and all methods were carried out in accordance with relevant guidelines and regulations. The trial will be a single-center, pragmatic, double-blind, cluster-randomized, placebo-controlled study. Adult patients presenting for an elective abdominal surgery through the colorectal, surgical oncology, or large ventral hernia ERAS services at a major academic institution will be eligible for enrollment. Major deviations from the standard ERAS protocols will be excluded, including the inability/patient refusal of a regional or neuraxial nerve block, a direct admission of an intubated patient to the Intensive Care Unit (ICU) from the operating room, and abortion of the planned surgical procedure (Table 1). Patients will be randomized to receive either a blinded ketamine or saline placebo administered intraoperatively as a bolus and infusion, followed by a postoperative infusion for up to 48 h. All other elements of the ERAS pathways will remain the same between the two groups.

**Table 1:** Inclusion and exclusion criteria for the IMPAKT ERAS trial

**Inclusion criteria**
Adult patients undergoing an elective major abdominal surgery within a colorectal, surgical oncology, or ventral hernia enhanced recovery pathway
**Exclusion criteria**
• Participant declines to consent for participation
• Contraindications to, or patient refusal, of standard enhanced recovery elements (e.g. nerve block)
• Surgery performed on a weekend day (non-elective)
• Direct postoperative admission from the operating room to the Intensive Care Unit with an endotracheal tube in place
• Cancellation or abortion of the planned surgical procedure
• Perioperative clinician deems the patient not appropriate for inclusion

### Pragmatic features (PRECIS Table 2)

**Table 2:** Pragmatic design features of the IMPAKT ERAS Trial

**PRECIS domain(s)**	**Assessment**
Eligibility criteria	This study will enroll all eligible major abdominal surgery patients within existing enhanced recovery pathways (colorectal, surgical oncology, & ventral hernia procedures) at a single academic medical center with few exclusions
Recruitment path	The IRB has approved a modified research consent process for this study, which leverages a shortened consent form. Eligible patients will be recruited and consented by a physician member of the Perioperative Medicine team during the analgesic nerve block consent process
Setting	Care will occur at a single, tertiary, academic medical center
Organization intervention	Usual enhanced recovery pathway care will be administered for patients by clinical staff. A blinded study medication containing either ketamine or saline placebo will be initiated as a bolus at the induction of anesthesia and maintained as a postoperative infusion for up to 48 h
Experimental and comparison interventions – delivery	The OR pharmacy will prepare the blinded study medications and will oversee the randomization schedule in variable week blocks
Experimental and comparison interventions – adherence	The lead study pharmacist reviews weekly reports of study drug administration to patients. Cross over events are identified by these reports. Adherence to the Enhanced Recovery Pathways is consistently monitored by a clinical dashboard that reports data in real-time. Early discontinuation of a study drug order requires providers to indicate a reason for cessation, which is captured in the EMR (e.g. side effects, early discharge anticipated)
Follow-up	In-hospital outcomes, including side effects and adverse outcomes, are monitored every 6 months by an independent Data Safety and Monitoring Board
Primary trial outcome	All outcomes of this study are pragmatically designed to be readily obtained within the clinical EMR. The primary outcome is the hospital length of stay, defined as the time from start of surgery until hospital discharge. Secondary outcomes, including adverse perioperative events, opioid requirements, frequency of antiemetic administrations, and postoperative nasogastric tube placement are also pragmatically available through the EMR
Analysis of primary outcome	All participants who are randomized will be included in the intention-to-treat statistical analysis, according to group assignment

*PRECIS* Pragmatic Explanatory Continuum Indicator Summary, *IRB* Institutional Review Board, *OR* operating room, *EMR* electronic medical record

The IMPAKT ERAS trial is supported by the Learning Healthcare System (LHS) at VUMC, which provides a supportive platform to integrate clinical practice with pragmatic research principles. The IMPAKT ERAS trial was intentionally designed to function within the constraints of routine clinical care at our institution where perioperative ketamine infusions have been utilized as a core component of our ERAS bundles since 2016. Therefore, the study protocols will be performed by clinical anesthesia providers (faculty, nurse anesthetists, and anesthesiology residents) without a need for a team of dedicated research staff. All clinical members of the Perioperative Medicine Service are trained on the trial protocol and consent process, as well as given a model script. A monthly IMPAKT ERAS orientation is performed to provide a refresher and to train new clinical members who rotate onto the team.

Implementation of a modified consent process was critical to preserving our pragmatic design. Our Learning Healthcare System worked closely with the IRB to develop a process that would easily embed within routine clinical workflow, yet also maintain patient autonomy and meet the standard regulatory elements of informed consent. As our research procedures were deemed no greater than minimal risk, some alterations of the informed consent process were permitted. The IRB approved an abbreviated, 1-page consent form that requires the participant’s written signature; however, an additional witness signature from the person obtaining consent is not necessary. Furthermore, the consent form remains with the nerve block consent and is uploaded to the patient’s EMR rather than requiring physical collection by the Principle Investigator (PI).

These modifications to the consent process were intentionally and carefully planned in close collaboration with our local IRB. The IMPAKT ERAS trial is the first study at our institution to implement this modified consent process. We believe this is in an important and novel aspect of our protocol that will serve as a platform for future pragmatic studies at our institution, and perhaps other centers at large.

### Interventions

In congruence with the clinical pharmacy’s current process of batching the production of ketamine infusions, randomization will occur in one-week cluster format, scheduled and tracked by the study pharmacist. All patients enrolled within a cluster will receive the same intervention (ketamine or saline placebo), with the choice being determined by a pre-generated random sequence. Both patients and their medical providers will be blinded to their randomization arm.

Participants assigned to the intervention arm will receive an intraoperative bolus of ketamine (0.5 mg/kg) followed by an infusion (5 mcg/kg/min) until fascial closure. A postoperative ketamine infusion (2.5 mcg/kg/min) will begin in the post-anesthesia recovery unit and continue for up to 48 h; however, it may be discontinued earlier for intolerable side effects, adverse events, or in preparation for patient discharge. Patients assigned to the placebo arm will receive an equivalent volume of saline for the bolus and infusions (Table 3). All other elements of the standard ERAS protocol will remain unchanged in both arms (Tables 4 and 5). The Anesthesia Perioperative Medicine Consult Service rounds daily on all ERAS patients to provide clinical care under the guidance of established protocols, including an algorithmic and consistent approach to the treatment of breakthrough pain. This team is the primary service to address pain and nausea, and a member of this team is available in-house 24 h per day, seven days per week (24/7).

**Table 3:** Ketamine and placebo interventions for the IMPAKT ERAS trial

**Intravenous Ketamine Intervention**
Intraoperative:
0.5 mg/kg bolus at anesthetic induction followed by intraoperative infusion of 5 mcg/kg/min until fascial closure, up to a maximum of 100 kg
Postoperative:
Infusion of 2.5 mcg/kg/min begun in PACU and continued for 48 h postoperatively or until discontinued, up to a maximum of 100 kg
**Placebo Intervention**
Intraoperative:
Placebo normal saline bolus and infusion of an equivalent volume mirroring the ketamine intervention
Postoperative:
Placebo normal saline infusion of an equivalent volume mirroring the ketamine intervention, continued for 48 h postoperatively or until discontinued

*mg* milligrams, *kg* kilogram, *mcg* micrograms, *min* minute, *PACU* postanesthesia care unit

**Table 4:** Multimodal analgesic protocol for major intra-abdominal surgery ERAS care pathways included in the IMPAKT ERAS trial at Vanderbilt University Medical Center

**Preoperative multimodal analgesic regimen:**
Acetaminophen: 1000 mg PO 1 h before OR time
• Reduce to 650 mg PO if < 70 kg
• avoid if Child score Class C liver disease
Gabapentin: 100–300 mg PO 1 h before OR time
• Reduce to 100 mg PO in patients > 65y
• Consider not giving or reducing to 100 mg PO in patients > 75y
• For those on home gabapentin, ensure that 100–150% of home dose was taken on AM of surgery (either at home or in preop holding area)
Regional Analgesia:
Mini-laparotomy or hand-assisted laparoscopy:
• Truncal nerve blocks – bilateral transversus abdominis plane (TAP) and rectus sheath blocks
◦ Ropivacaine 0.25% + dexamethasone 4 mg (30-45 mL/side total)
Ostomy takedown/creation with unilateral incision
• Bilateral TAP blocks (if ostomy above umbilicus, consider adding rectus sheath)
◦ Ropivacaine 0.25% + dexamethasone 4 mg (30-45 mL/side total)
Laparotomy:
• Thoracic epidural catheter (TEC) with infusion starting at 8 mL/hr with 3 mL/15 min patient controlled epidural analgesia (PCEA)
• 0.1% ropivacaine + 10 mcg/mL hydromorphone
◦ Adjustment to rate and concentration determined by clinical outcomes in pain coverage and hemodynamics
**Intraoperative multimodal analgesic regimen:**
Opioids: No induction opioids, and minimize opioid use during anesthetic
• If necessary, use esmolol for heart rate control and an anti-hypertensive of choice for BP control
• Assess need for opioid upon emergence
◦ Utilize methadone 5 mg IV bolus q5-10 min as first-line prior to other opioids
Thoracic epidural: utilize if present, consider bolus with 0.125- 0.25% bupivacaine prior to incision
Lidocaine infusion: 1.5 mg/kg bolus with induction, then 2 mg/min drip from induction to case end
Ketamine infusion: 0.5 mg/kg IV bolus with induction, infusion of 5mcg/kg/min IV after induction until fascia closure (up to 100 kg)
Ketorolac: 30 mg IV at fascia closure
◦ reduce to 15 mg IV if > 65y, CrCl < 30, or patient weight < 50 kg
◦ consider avoiding for h/o renal dysfunction or GI bleed
**Postoperative multimodal analgesic regimen:**
Lidocaine infusion for 24 h (continued from PACU or after TEC removed)
• 1 mg/min IV if < 70 kg
• 1.5 mg/min IV if 70–100 kg
• 2 mg/min IV > 100 kg
Ketamine Infusion for 48 h (continued from PACU)
• 2.5 mcg/kg/min infusion (use patient’s body weight, up to a max of 100 kg)
• Consider prolonging infusion for continued Nil Per OS or uncontrolled pain
• Contraindications: increased intracranial pressure, increased intraocular pressure
Acetaminophen 1000 mg PO Q8hr starting POD 0 until discharge
• Reduce to 650 mg PO Q6h if < 70 kg
• Reduce to 500 mg Q8h for liver disease
• Don’t use if Child Class C liver disease
Gabapentin 300–600 mg PO q8h starting POD 0 until discharge.
Use lower dose for > 65y or if patient having significant sedation/dizziness
Dose/frequency should be adjusted based on renal function:
• CrCl > 60 ml/min: 300-1200 mg TID
• CrCl > 30–59 ml/min: 200-700 mg BID
• CrCl > 15-29 ml/min 200-700 mg once daily
• CrCl < 15- reduce dose in proportion to CrCl ~ 100–300 mg once daily
• ESRD requiring hemodialysis- dose based on CrCl, plus single supplemental dose of 125-250 mg after dialysis
Ketorolac: 30 mg IV Q6h × 3 days
• Reduce to 15 mg IV Q6h in patients > 65y, CrCl < 30, or weight < 50 kg
Opioid PRN
• Consider lowest possible dose and frequency for pain control
• For opioid naïve patients:
◦ First line: tramadol 50 mg q 4–6 H PRN (max 400 mg/24 h), for pain > 4/10
◦ Second line: oxycodone 5 mg PO Q4 PRN pain > 4/10
◦ Breakthrough pain: consider hydromorphone IV PRN bolus
• For patients on chronic opioid therapy: ensure meeting 100–125% of home oral dose, with goal of avoiding significant opioid escalation
Regional Analgesia:
• Truncal nerve blocks can be repeated on POD 1 if uncontrolled pain is determined to be incisional
• TEC, if present: continue with infusion at 8 mL/hr of 0.1% ropivacaine + 10 mcg/mL hydromorphone with 3 mL/15 min PCEA
◦ Adjustment to rate and concentration determined by clinical outcomes in pain coverage and hemodynamics
◦ Re-evaluate each day for the necessity of the epidural up to 5 days. It is typically removed on the day prior to anticipated discharge to ensure adequate pain control on an oral regimen

*ERAS* enhanced recovery after surgery, *mg* milligrams, *PO* per os, *OR* operating room, *kg* kilograms, *y* years, *AM* Ante Meridiem (morning), *mL* milliliters, *hr* hour, *mcg* micrograms, *min* minute, *BP* blood pressure, *IV* intravenous, *q* quaque (every), *CrCl* creatinine clearance, *h/o* history of, *GI* gastrointenstinal, *PACU* postanesthesia care unit, *POD* postoperative day, *h* hour, *ESRD* end stage renal disease, *PRN* pro re nata (as needed), *hrs* hours

**Table 5:** Anti-emetic protocols for major intra-abdominal surgery ERAS care pathways included in the IMPAKT ERAS trial at Vanderbilt University Medical Center

ERAS Perioperative Anti-Emetic Regimen
Pre and Intraoperative Strategies:
• Number of PONV prophylaxis agents should equal the number of Apfel Risk Factors
• Avoid use of volatile agents: Total Intravenous Anesthesia (TIVA) with propofol is preferred
• Intraoperative Pharmacologic Agents:
Dexamethasone: 8 mg IV after induction unless given in TAP blocks
Ondansetron: 4 mg IV given 30 min prior to emergence
Haloperidol: 1 mg IV given during skin closure, reduce to 0.5 mg IV in patients > 65 years
Postoperative Pharmacologic Agents:
Ondansetron: 4 mg IV/PO q 6 h PRN (write:1st line for nausea and vomiting)
Haloperidol: 0.5 – 1 mg IV PRN q 4–6 h PRN (write: 2nd line after ondansetron)
Scopolamine patch (write: 3rd line option only if active PONV despite above)
Promethazine: 6.25–12.5 mg IV/PO q 4 h PRN (write: 4^th^ line option)

*ERAS* enhanced recovery after surgery, *PONV* postoperative nausea and vomiting, *mg* milligrams, *IV* intravenous, *TAP* transversus abdominis plane, *PO* per os, *q* quaque (every), *h* hours, *PRN* pro re nata (as needed)

### Outcomes

Hospital LOS, defined as the time between anesthesia start and patient discharge, was chosen as the primary outcome because it is an objective marker of functional recovery after surgery. It is the customary outcome measured when evaluating the overall efficacy of ERAS protocols to facilitate faster recovery compared to routine care. Moreover, LOS represents a metric that is important to patients and families, physicians, and healthcare systems. Secondary outcomes include the incidence of adverse perioperative events, such as rapid response activation and escalation of care to an ICU. We will also investigate the frequency of antiemetic administrations and placements of postoperative nasogastric tubes for severe ileus.

The analgesic impact on total opioid consumption, measured as morphine milligram equivalents of opioid use until hospital discharge, will be investigated as a secondary outcome, as opioid minimization has been identified as an important contributor to the success of an ERAS program. Within our institution’s abdominal ERAS programs, perioperative median morphine milligram equivalents (MMEs) are intentionally minimized. Therefore, it is possible that a further reduction in opioids may of be negligible benefit, and perhaps the side effect profile of ketamine impairs recovery rather than promotes it. The incidence of side effects will be compared across the two arms by examining the documented reasons for and frequencies with which the investigational drug infusions are discontinued early, prior to the 48-h duration as intended.

### Sample size planning

ERAS pathways for major abdominal surgery were established at VUMC in 2013. Prior to the addition of perioperative ketamine infusions to these protocols in 2016, the median[interquartile range] hospital length of stay was 4.3[3.3–6.4] days. To determine the sample size needed for sufficient power to detect a meaningful difference, the sample size calculation assumed 85% power, a two-sided type I error rate of 5%, and a 10%, reduction in hospital LOS. The sample size was calculated in R using the method described by Li and colleagues [14]. The calculation estimated the within period correlation from preliminary data concerning the LOS for major abdominal surgeries to be about 0.014. Given these assumptions, about 757 patients would need to be included in each arm. An increase in sample size was deemed necessary to accommodate for the two post-randomization exclusions that are pre-specified. Accordingly, the total target enrollment will be 1,544 participants over a 2 year period.

### Recruitment

Adult patients (≥ 18 years) presenting to VUMC on a weekday for an elective, major abdominal surgery within an established ERAS protocol are eligible for enrollment. Per usual care, patients are approached on the day of their surgery by a physician member of the Anesthesia Perioperative Medicine Consult Service who describes our care goals and multimodal pathways, and then obtains consent to perform a regional or neuraxial nerve block. Regional blocks are offered for laparoscopic and small open incisions (< 10 cm) and include abdominal wall blocks (e.g. rectus sheath, transversus abdominis plane). Neuraxial anesthesia (thoracic epidural catheter) is offered for patients planned to have open procedures. The IRB approved a modified research consent process for the trial with research consent also obtained when clinical consent for the regional or neuraxial nerve block is obtained. If the patient elects to participate, written consent will be collected by the Anesthesia Perioperative Medicine Consult Service physician and uploaded to the EMR.

### Allocation

Simple randomization will occur in two and four-week cluster format, with the treatment arm assigned by a random sequence pre-generated by our study statistician. The randomization list was distributed to our study pharmacist who provided oversight to pharmacy operations. Other than the study pharmacist, the rest of the study team has remained blinded to the randomization scheme and study assignments. Weekly batches of the appropriate study drug are prepared by the Operating Room pharmacy in generic infusion bags with a non-identifying study label. The study drug is stocked in a refrigerated Omnicell located in preoperative holding, and the contents are replaced with the new batch each week. The lead study pharmacist reviews weekly reports of study drug dispersal and administration to identify cross over events.

### Data analysis and management

Initial analysis will use descriptive statistics and data visualizations to both identify and address spurious values, and to characterize the study cohort. Characteristics will be described overall and grouped by study arm, without a plan to compare the study arms with statistical tests. Categorical variables will be described using frequencies and proportions; continuous variables will be described using means and standard deviations as well as medians and IQRs. Missingness will be recorded for each variable.

The primary outcome is hospital LOS, measured in days. It is expected that LOS will have a skewed distribution, and thus non-parametric methods are preferred. However, it is not possible to consider the clustering or baseline covariates using a Wilcoxon test. Therefore, a proportional odds regression model will be used. The model will include baseline characteristics such as age, body mass index (BMI), smoking status, opioid use, and type of surgical procedure. Multiple imputation based on predictive mean matching will be used for missing covariates. Continuous variables will be modeled flexibly using cubic splines. Clustering will be taken into account using a mixed-effects model. If there are patients included twice because they underwent two separate hospitalizations for elective major abdominal surgeries, those patients will also be included as a random effect. The common odds ratio from the model will be estimated; this quantity can be interpreted similar to the concordance probability and is robust to the proportional odds assumption. Model-assisted estimates of mean and median length of stay will also be reported. Bootstrapping will be used to generate appropriate confidence intervals.

The primary analysis will be an intention to treat analysis including all randomized participants that meet the inclusion/exclusion criteria, analyzed according to group assignment. If a participant or provider withdraws the participant from the study following study drug administration, the participant will receive care at the provider’s discretion, but will remain in the intention to treat statistical analysis.

The secondary outcomes will be explored in a similar way. The proportional odds model will be used to evaluate differences in opioid use in the hospital. Occurrence of events will be reported as rates with confidence intervals. Logistic regression will be used to compare the odds of events between groups.

In all statistical modeling, emphasis will be placed on effect sizes over p-values. In addition, continuous variables will be modelled flexibly using cubic splines. Differential treatment effects will be explored by examining the interaction between the treatment indicator and the putative subgrouping variable. Effects of treatment by sex will be analyzed. As before, continuous variables will not be categorized, and any interactions will be evaluated as continuous variables. Subgroup analyses will not occur in reporting the main results of this trial unless there is evidence of a differential treatment effect on interaction testing.

### Monitoring

Patients enrolled in the trial are closely followed by their surgical services and the Anesthesia Perioperative Medicine Consult Service, both of which round daily and have providers available in the hospital at all times. Any deviations from the protocol or unanticipated events are identified promptly and immediately reported to the Principal Investigator (PI), who is responsible for reporting the events to the IRB.

**Fig. 1:**
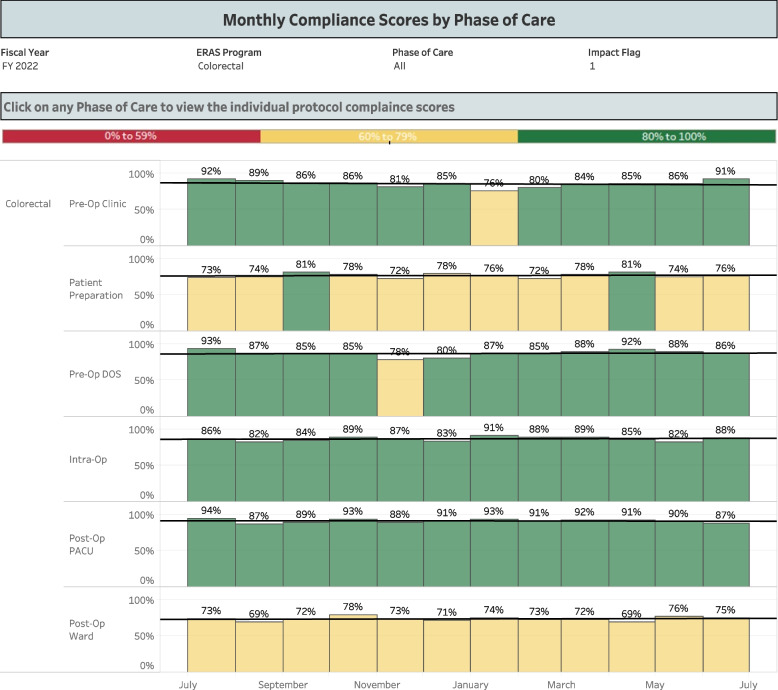
Compliance dashboard monitoring our institution’s adherence to the colorectal ERAS protocol, by phase of care

**Fig. 2:**
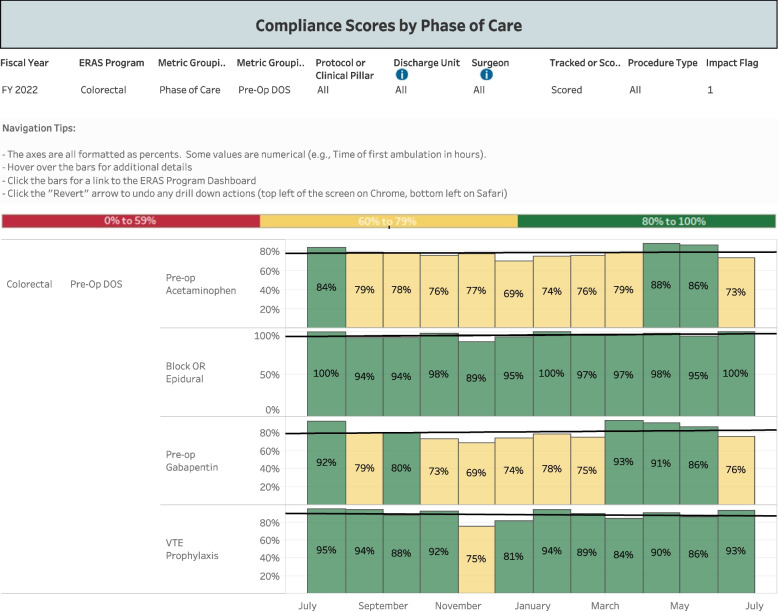
Compliance dashboard monitoring our institution’s adherence rates to preoperative elements of the colorectal ERAS protocol

The ERAS protocols at VUMC provide guidelines for the clinical management of acute perioperative pain. Adherence to the protocols is vital to the integrity of the trial, as all elements of the pathway (apart from ketamine administration) are to remain comparable between groups. A dedicated Anesthesia Perioperative Medicine Consult Service offers consistency in applying these ERAS principles. Furthermore, adherence to ERAS elements is regularly monitored through an electronic dashboard that provides real-time analysis of patient data and ordering practices (Figs. 1 and 2).

### Data and safety monitoring

A Data Safety and Monitoring Board (DSMB) of three independent, unaffiliated, multidisciplinary specialists meet at least every 6 months to ensure that the rights and well-being of participants are protected and the reported data are accurate and complete. At each meeting, DSMB members are apprised of the accrual of subjects, fidelity to the protocol, and reports of serious adverse events and unanticipated adverse events. The DSMB is also unblinded to the results of the outcomes at their meetings during a closed session. The DSMB provides advisory council to the PI and has authority to recommend halting the trial if continuation is deemed unsafe. The final recommendations of the DSMB will be immediately reported to the IRB.

### Ethics

Ketamine is an analgesic that has been proven to reduce opioid consumption after surgery. It also has a role in reducing the development of chronic pain. Compared to opioids, ketamine has a favorable side-effect profile, including the maintenance of respiratory and cardiovascular stability. Ketamine does not act at the mu opioid receptor, and therefore is not associated with nausea, itching, constipation, and tolerance. We have been safely utilizing perioperative ketamine at VUMC for several years as part of our enhanced recovery pathways. The IMPAKT ERAS study design does not involve the introduction of a new treatment or intervention, but rather a structured, yet pragmatic, investigation of the impact of currently administered medical care, with an intent to leverage data that is already collected as part of routine clinical care to use as outcomes. The IMPAKT ERAS trial has been deemed minimal risk above clinical risk by the IRB, allowing for alteration of the informed consent process.

#### Adverse reactions to ketamine

Adverse reactions and allergies to ketamine are routinely reviewed by both medical providers and pharmacy personnel prior to ordering and administering the drug. Ketamine is a controlled substance and is highly regulated, recorded, and tracked. Patients are evaluated daily at the bedside by our Anesthesia Perioperative Medicine Consult Service, and patients are screened for side effects of ketamine on daily rounds. Nurses are trained to immediately report any suspicion of an adverse ketamine reaction to a member of our team, who is available in the hospital at all times.

#### Selection of patients

Only patients receiving care through our enhanced recovery pathway (which includes the standard administration of ketamine) whose provider agrees is appropriate for inclusion and who signs the research consent will be eligible for participation.

#### Ineffective pain control

Patients with breakthrough pain will receive routine treatment, including opiate administration if needed, at the discretion of medical providers through the guidance of our analgesic protocols.

#### Breach of confidentiality

One of the risks specific to the research study is potential breach of confidentiality. The investigators have access to the medical data as part of their job standing. To minimize this risk, all database work will be performed on VUMC approved and password protected servers that are physically located in the VUMC computing environment and maintained by VUMC security standards. Only the PI and key study personnel will have access to study specific non de-identified data.

## Dissemination

The results of the IMPAKT ERAS trial will be submitted for presentation at local, national, and international medical conferences, as well as academic institutions through visiting and invited lectureships. Furthermore, we aim to publish our results in a high-impact, scientific journal that will distribute the data to a broad clinical audience.

## Conclusions

The IMPAKT-ERAS trial is a pragmatic study investigating the individual contribution of adding perioperative ketamine infusions to an established ERAS pathway. Ketamine, as a sole agent, is a well-established analgesic with opioid-sparing potential. However, in a multimodal setting with low opioid utilization, it is unclear if ketamine has the ability to speed recovery without introducing an untoward side effect profile. The IMPAKT ERAS trial will yield new information on the effects of perioperative ketamine on functional recovery following major abdominal surgery within ERAS programs.

## Data Availability

Not applicable at this time.

## Abbreviations

24/7: 24 Hours per day, seven days per week
BMI: Body mass index
DSMB: Data Safety and Monitoring Board
EMR: Electronic medical record
ERAS: Enhanced recovery after surgery
ICU: Intensive Care Unit
IMPAKT ERAS: IMpact of PerioperAtive KeTamine on​ Enhanced Recovery from Abdominal Surgery
IQR: Interquartile range
IRB: Investigational Review Board
kg: Kilogram
LHS: Learning Healthcare System
LOS: Length of stay
mcg: Microgram
mg: Milligrams
min: Minute
MME: Morphine milligram equivalent
NMDA: N-methyl-D-aspartate
PI: Principal Investigator
VUMC: Vanderbilt University Medical Center
